# General Study and Gene Expression Profiling of Endotheliocytes Cultivated on Electrospun Materials

**DOI:** 10.3390/ma12244082

**Published:** 2019-12-06

**Authors:** Alena O. Stepanova, Petr P. Laktionov, Anna V. Cherepanova, Vera S. Chernonosova, Georgiy Yu. Shevelev, Ivan A. Zaporozhchenko, Alexander M. Karaskov, Pavel P. Laktionov

**Affiliations:** 1 Laboratory of Biomedical Technologies, Meshalkin National Medical Research Center, Ministry of Health of the Russian Federation, Rechkunovskaya str. 15, 630055 Novosibirsk, Russia; a_cher@niboch.nsc.ru (A.V.C.); vera_mal@niboch.nsc.ru (V.S.C.); ivanzap@niboch.nsc.ru (I.A.Z.); meshalkin@meshalkin.ru (A.M.K.); lakt@niboch.nsc.ru (P.P.L.); 2 Laboratory of Molecular Medicine, Institute of Chemical Biology and Fundamental Medicine, Siberian Branch of the Russian Academy of Sciences (ICBFM SB RAS), Lavrentiev ave. 8, 630090 Novosibirsk, Russia; 3 Department of the Regulation of Genetic Processes, Laboratory of Genomics, Institute of Molecular and Cell Biology, Siberian Branch of the Russian Academy of Sciences (IMCB SB RAS), Lavrentiev ave. 8/2, 630090 Novosibirsk, Russia; laktionov@mcb.nsc.ru; 4 Department of Natural Sciences, Epigenetics Laboratory, Novosibirsk State University, Pirogova str. 2, 630090 Novosibirsk, Russia; 5 Laboratory of Biomedical Chemistry, Institute of Chemical Biology and Fundamental Medicine, Siberian Branch of the Russian Academy of Sciences (ICBFM SB RAS), Lavrentiev ave. 8, 630090 Novosibirsk, Russia; shevelev@niboch.nsc.ru

**Keywords:** electrospinning, 3D matrices, polycaprolactone, vascular grafts, endothelization, RNA sequencing

## Abstract

Endothelization of the luminal surface of vascular grafts is required for their long-term functioning. Here, we have cultivated human endothelial cells (HUVEC) on different 3D matrices to assess cell proliferation, gene expression and select the best substrate for endothelization. 3D matrices were produced by electrospinning from solutions of poly(D,L-lactide-co-glycolide) (PLGA), polycaprolactone (PCL), and blends of PCL with gelatin (Gl) in hexafluoroisopropanol. Structure and surface properties of 3D matrices were characterized by SEM, AFM, and sessile drop analysis. Cell adhesion, viability, and proliferation were studied by SEM, Alamar Blue staining, and 5-ethynyl-2’-deoxyuridine (EdU) assay. Gene expression profiling was done on an Illumina HiSeq 2500 platform. Obtained data indicated that 3D matrices produced from PCL with Gl and treated with glutaraldehyde provide the most suitable support for HUVEC adhesion and proliferation. Transcriptome sequencing has demonstrated a minimal difference of gene expression profile in HUVEC cultivated on the surface of these matrices as compared to tissue culture plastic, thus confirming these matrices as the best support for endothelization.

## 1. Introduction

Endothelization of the surfaces of cardio-vascular implants is necessary for their long-term functioning. Seeding of the surfaces with endothelium is equally as important as mechanical compliance and the capability for long-term functioning without loss of stiffness [1]. Ineffective endothelization is considered as the primary cause of low long-term patency of small diameter vascular grafts (VG) and calcification of valve flaps [2,3]. A number of studies have demonstrated that the absence of functional endothelial layer can reduce hemocompatibility, induce blood clotting and intensive neointima growth in the lumen of small diameter VG [4]. Malformation or malfunctioning of the endothelial layer can induce systemic or local inflammation with subsequent fibrosis or calcinosis of the damaged area [5]. Endothelization is affected not just by the surface but also the structural properties of the material. Transcytosis and bidirectional permeable barrier function must be physically enabled by the permeability of the scaffold. Deformation of the material in response to pulse wave should be similar to natural arteries to ensure the fluid flow within the material and promote transport function of endothelial cells [6,7]. Suitable 3D matrices must have perforating pores and a surface that is capable of supporting cell adhesion, proliferation, establishment of cell-to-cell contacts, and formation of a functional endothelial cell layer [8]. 

A number of studies have recognized the suitability of 3D matrices produced by electrospinning (ES) from synthetic or natural polymers and their blends for production of cardiovascular implants [9,10,11]. These 3D matrices consist of fibers with a diameter ranging from tens of nanometers to several micrometers, and can be composed of different layers, for example, an inner smooth layer or layers with different degree of cell permeability [12]. ES additives, including drugs and protein molecules, can be introduced into the fibers in a simple and robust manner [13,14]. Previously, such matrices were successfully used in the production of VG (e.g., Nicast AVflo, [15]) and cardiac valves [16]. Different polymers can be used for VG production by ES. Polycaprolactone (PCL), in particular, was extensively studied in this context, both as a sole component of matrices and blended with different proteins [17,18,19]. In the latter case, the proteins were exposed at the fiber surface, free to interact with cellular receptors, thus facilitating cell adhesion and promoting growth [20]. 

To study the endothelization of 3D matrices, several types of cells can be used, including primary endothelial cells obtained from arterial endothelium (human coronary artery endothelial cells, human aortic endothelial cells, human pulmonary artery endothelial cells), microvascular endothelial cells (human dermal microvasculature endothelial cells, human pulmonary microvasculature endothelial cells, human brain microvasculature endothelial cell), circulating endothelial progenitor cells or human umbilical vein endothelial cells, as well as transformed endothelial cells [21,22,23,24]. Use of human over animal endothelial cells is advisable because of repeatedly observed inadequacy of animal experimental models, convenience of data analysis and direct applicability of the results [25]. Since primary cells tend to lose their phenotype during long-term cultivation, a renewable source of cells is needed for systematic study [26]. Cells from different regions of cardiovascular systems have distinct properties and arterial endothelial cells are best-suited for the task but are not easily obtained due to ethical barriers. So far, most research was performed using human umbilical vein endothelial cells (HUVEC) [22]. These cells have a reasonably good proliferative capacity, and, although different from arterial endothelial cells (partially due to cultivation in oxygen-rich atmosphere), can still be considered phenotypically and functionally normal endothelial cells in the first few passages [27]. 

A common strategy to characterize the functional status of cells cultivated on 3D matrices used to involve an analysis of several cellular antigens by immunohistochemistry [28,29,30]. Obviously, a functional state of cells cannot be fully characterized by the expression of a small set of proteins and thus assessment of cell transcriptome by microarrays or RNA sequencing is becoming increasingly more prevalent [31,32,33,34,35]. At that, compared to microarray, sequencing is considered to be a more robust, accurate, and unbiased method of gene expression profiling [36].

Here, we studied the functional state of HUVEC cultivated on different 3D matrices produced by ES from poly(D,L-lactide-co-glycolide) (PLGA), PCL and blends of PCL with gelatin (Gl) by gene expression profiling on a HiSeq 2500 platform. The aim of the study was to perform hypothesis-independent evaluation of the physiological state of the cells in respect to matrix composition and structure. To our knowledge, a comparative study of gene expression profiles of endothelial cells cultivated on electrospun 3D matrices has not been previously reported elsewhere.

## 2. Materials and Methods

### 2.1. Fabrication of Electrospun Matrices

The electrospinning solutions were prepared in 1,1,1,3,3,3-hexafluoroisopropanol using PLGA 50:50 (Lactel, B6010-1, Birmingham, AL, USA), pure PCL (Aldrich, 440744, St Louis, MO, USA) or PCL blended with Gl (PCL-Gl) from porcine skin (Sigma, G-2500, Switzeland). 3D matrices with a thickness of 120–150 µm were produced from 15% (*w*/*v*) PLGA, 7% (*w*/*v*) PCL or 5% (*w*/*v*) PCL-Gl (9:1 *w*/*w*) solutions on an NF-103 (MECC CO., LTD., Ogori-shi, Fukuoka, Japan) electrospinning device using a blunt-end surgical needle 27G (0.4 mm inner diameter). Fibers were electrospun onto a rotating drum collector (diameter 60 mm, length 35 mm) under the following conditions: feed rate 1–1.15 mL/h, capillary-collector distance 19–20 cm, voltage 18.5–24 kV, drum collector rotation speed 300 rpm, humidity 25–35%. After fabrication, 3D matrices were removed from the collector, incubated under vacuum for 12 h and stored in sealed zip-lock polyethylene containers at 4 °C. 

### 2.2. Analysis of Matrix Surface Structure and Properties 

The microstructure of the matrix surface was studied using scanning electron microscopy (SEM), (JSM-6460 LV, Jeol, Tokyo, Japan) as described previously [20] and atomic force microscopy (AFM) with Multimode 8 (Bruker, Germany) using NSG10-DLC diamond-like carbon NSG series AFM cantilever (NT-MDT, Zelenograd, Russia) in tapping mode. Fiber diameter and pore size were calculated from SEM images according to ISO 7198:1998 using AxioVision 4.8 software (Carl Zeiss, Gottingen Germany). The contact angle was determined with a Drop Shape Analyzer DSA25 (Kruss GmbH, Germany) using water as a solvent. Drop volume was set to 1 µl and camera speed was 160 frames per second as recommended by the manufacturer. The contact angle was calculated from 4 replicates for each type of 3D matrices.

### 2.3. Treatment of Matrices with Glutaraldehyde

To obtain glutaraldehyde-treated PCL-Gl (PCL-Gl-glu), PCL-Gl matrices were incubated in 0.05 M NaHCO_3_ (pH 9.1) in a horizontal shaker for 10 min to moisten the material and then treated by 2% glutaraldehyde (Aldrich, St Louis, MO, USA) in 0.05 M NaHCO_3_ solution for 2 h at room temperature. After the incubation, matrices were washed thrice with 0.05 M NaHCO_3_ (pH 9.1) for 5 min. Remaining free reactive groups were blocked by incubating the matrices in 10 mM glycine (ICN, Aurora, Ohio, USA) solution (pH 9.1) for 30 min followed by incubation in freshly prepared 0.2 mg/mL NaBH4 for 20 min. After the incubation, matrices were thoroughly washed with three changes of pyrogen-free deionized H_2_O (arium 611VF, Sartorius, Gottingen, Germany) with 0.5% glycerol (Aldrich, St Louis, MO, USA) and air-dried. The obtained PCL-Gl-glu matrices were stored in sealed zip-lock polyethylene containers at 4 °C.

### 2.4. Isolation, Cultivation and Characterization of Human Umbilical Vein Endothelial Cells

Human umbilical vein endothelial cells (HUVEC) were isolated and cultivated as described previously with modifications [37,38]. Briefly, an umbilical vein was washed with 50 mL of phosphate buffer (PBS), then with 20 mL of collagenase IV buffer (1.5 mM HEPES, 14 mM NaCl, 0.4 mM KCl, 0.12 mM CaCl_2_, 0.04 mM MgSO_4_, 0.76 mM D-glucose, pH 7.4), and incubated for 15 min at 37 °C with 0.1% collagenase IV. After incubation, the solution of 0.1% collagenase IV with detached cells was collected, the vein was additionally washed with 20 mL PBS, and both solutions were combined and centrifuged at 800 g for 10 min to pellet cells. Supernatant was discarded, cell pellet was resuspended, and cells were cultivated in Petri dishes covered with 0.5% gelatin solution in IMDM culture medium (Gibco, Aucland, New Zealand) with 10% FBS (Gibco, USA) and antibiotics. On the next day, adherent cells were carefully washed from blood cells with IMDM and further cultivated in fresh IMDM with 10% FBS (passage 0). The cells were passaged once per 3–4 days after 70–80% confluence was reached. Following 12 h after seeding, adherent cells were carefully washed with fresh culture medium. To dissociate cells, a 0.1% solution of collagenase IV was used. 

To cultivate cells on the surface of matrices, second passage HUVEC were seeded in 8-well Nunc Lab-Tek Chamber Slide System (Invitrogen, Carlsbad, CA, USA) (10^4^ cells per well). After reaching the 50% confluence, cells were washed with IMDM, fixed with 3.7% paraformaldehyde in IMDM, and permeabilized with 0.1% Triton X-100. Sections were incubated for 2 h at 37 °C with primary antibodies against VEGF-A (ab1316), VE-Cadherin (ab166715), von Willebrand Factor (ab68545) (Abcam, Cambridge, UK), CD31 (PECAM1) (M082301-2) (Dako, Agilent, Santa Clara, CA, USA), then incubated for 1 hour at 37 °C with Alexa 488-conjugated secondary antibodies (ab150077 and ab1500117, Abcam, Cambridge, UK). After staining with TRITC/rhodamine-conjugated phalloidin (Sigma-Aldrich, Saint Louis, MO, USA) for F-actin, glass slides were covered with ProLong Gold antifade mounting medium with DAPI (Invitrogen, Carlsbad, CA, USA) for staining of the nuclei, mounted with a coverslip and analyzed using a confocal laser scanning microscope (LSM 510 UV Meta or LSM 780 NLO, Carl Zeiss, Gottingen Germany). Three laser lines at 405 nm (to detect cell nuclei stained with DAPI), 543 nm (to detect actin microfilaments stained with phalloidin-TRITC), and 488 nm (to detect Alexa Fluor 488 secondary antibodies) were used.

### 2.5. Assessment of Adhesion and Viability of Endothelial Cells on the Matrix Surface

To test cell adhesion and proliferation on 3D matrices, HUVEC were cultivated in tissue culture polystyrene (TCPS) 48-well culture plates. Disks were excised from matrices by die cutting, placed in the wells of a 48-well plate, and fixed at the bottom by polytetrafluoroethylene O-rings (outer and inner diameters, 10 and 8 mm). The discs were pre-incubated in culture medium for 2 h to completely moisten the matrices. Culture medium was removed and endothelial cells were seeded into the wells (2–20 × 10^3^ cells/well). Polytetrafluoroethylene rings were also installed in control wells to make the free bottom surface area even across all the wells. Following 48 h cultivation, the viability of endothelial cells was assessed using Alamar Blue (Invitrogen, Eugene, Oregon, USA) as described previously [20]. Matrices not seeded with cells were used as control for dye sorption on the material, and the surface of the 3D matrices (after O-ring installation) was taken into account in the further estimations. 

To evaluate the number of cells in S-phase that undergo active DNA synthesis, 5-ethynyl-2’-deoxyuridine (EdU) incorporation visualized by copper catalyzed click-reaction between alkyne and Alexa Fluor 488 azide was used (Invitrogen, Eugene, Oregon, USA). Cells were incubated with EdU for 2 h after 48 h seeding and treated as recommended by the manufacturer (Invitrogen Click-iT EdU Alexa Fluor 488 Imaging Kit). The number of green cells and total cells were calculated in 5 fields of view with an area of about 0.45 mm^2^ (30% of the surface area of the 3D matrix) using an Axiovert 200M microscope supplied with AxioVision 4.8 software (Carl Zeiss, Gottingen Germany).

3D matrices seeded with HUVEC were prepared for SEM as follows: after 48 h cultivation, culture medium was removed from the wells; matrices were washed twice with phosphate buffer, fixed with 2% formaldehyde in physiological saline solution for 30 min, washed thrice with H_2_O, and air-dried. Matrices were removed from the wells, sputter-coated with 10-nm gold film, and examined by SEM as described previously [20].

Endothelial cells from 3 biological donors were used for all functional tests.

### 2.6. Cultivation of HUVEC for Gene Expression Profiling and RNA Isolation

To evaluate the gene expression profile of HUVEC obtained, 2 biological donors were used. To prevent the accumulation of chromosome rearrangements in HUVEC after passages 4–5, especially when cells are dissociated with trypsin [22], only cells after passage 1 and dissociated with collagenase IV were used to study gene expression. To avoid log growth phase and normalize HUVEC cultivation between different 3D matrices, cells were seeded in higher density. Cells (7 × 10^4^) were seeded in the wells of a 24-well plate with or without 3D matrices secured at the bottom by polytetrafluoroethylene O-rings and cultivated as described previously (O-ring footprint ~1.2 cm^2^). After 48 h of cultivation culture medium was removed, matrices were washed with one change of phosphate buffer and transferred to tubes with 750 μl of TriZol (Life Technology, Carlsbad, CA, USA).

RNA was isolated using TriZol according to the protocol provided by the manufacturer. Samples contained from 1.5 to 3.5 μg RNA. RNA was precipitated by 70% alcohol with 40 µg of glycogen (Thermo Scientific, Lithuania), the pellet was dried, dissolved in RNA stable storage medium (Sigma-Aldrich, St Louis, MO, USA), and then dried on a vacuum rotary evaporator. RNA quality in all samples was confirmed by the Bioanalyzer system Agilent 2100 (Agilent Technologies, Waldbronn, Germany) immediately after isolation and prior to sequencing in Turku sequencing center (Finland).

### 2.7. RNA Sequencing and Data Analysis

RNA-Seq libraries were prepared with the Illumina TruSeq Stranded mRNA Sample preparation kit, and sequenced on the HiSeq2500 platform using single-end 50 bp sequencing. Library preparation and sequencing were performed at Finnish Functional Genomics Centre (FFGC, University of Turku, Turku Centre for Biotechnology, Turku, Finland). The quality of all libraries was assessed at the sequencing facility and was within appropriate range for all samples, except one HUVEC sample cultivated onto PLGA 3D matrices (Table S1). Raw data quality control was performed using the FastQC from FASTX-toolkit tool collection [39]. All RNA-Seq reads across all samples demonstrated appropriate Q30 per base sequence quality level and GC-content that follows a normal distribution with the peaks at the expected 40–60%. RNA-seq reads were aligned to NCBI GRCh38 genome using TopHat2 aligner v 2.0.14 (USA) [40]. Overall alignment rate for all samples, except PLGA matrices, was higher than 95% and percentage of uniquely mapped reads from all mapped reads was in the range of 82–90%. FeatureCounts tool from the Subread package was used to count reads mapped to genomic features [41]. Differential expression analysis was performed with DESeq2 [42]. Following criteria were used to consider genes as differentially expressed-FDR adjusted *p*-value < 0.05 and absolute value of log_2_ fold change (FC) > 1. To generate RPKM values normalized across all the samples, we used Cuffnorm tool v.2.2.1 (USA) using the geometric normalization method [43]. The data generated in this study were submitted in Gene Expression Omnibus (GEO), accession number GSE123045.

### 2.8. Statistical Data Analysis 

Microsoft Excel 2010 (Microsoft Corporation, Redmond, WA, USA) was used to manage and sort experimental data. Statistical analyses were performed using the statistical software package Statistica v.7.0 (StatSoft Inc., Tulsa, OK, USA).

## 3. Results and Discussion

### 3.1. Characterization of 3D Matrices

The matrices were prepared from PCL, PCL with Gl and PLGA by ES in conditions listed in Table 1. In the preliminary experiments, we have discovered that the efficacy of HUVEC adhesion to PCL-Gl 3D matrices positively correlated with Gl concentration up to 10%, but further increase had no significant effect on cell adhesion. XPS demonstrated that the concentration of Gl on the surface of the fibers in these matrices was no less than 21%, and most of the surface-exposed Gl was tightly bound to the fibers [44]. It was previously shown that, in 3D matrices produced from PCL with 10% HSA (w:w), ~20% of the protein was exposed at the surface while only 34.7% of HSA was exposed at the surface of the fibers produced from PCL with 30% HSA [20]. Excess surface Gl can dissociate from the fibers, potentially negatively affecting cell adhesion. Thus, in this study, we used 3D matrices made of PCL with 10% Gl.

**Figure 1:**
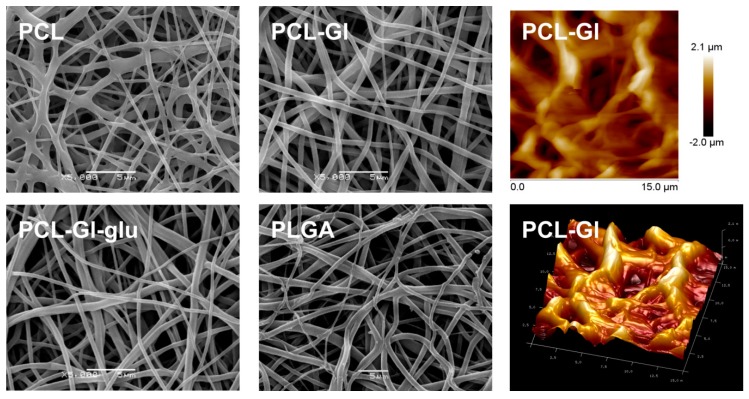
SEM images of 3D matrix surface. The right-most column shows AFM images of the surface of PCL-Gl matrices.

SEM showed that all matrices consisted of fibers with a diameter of approximately 1 μm and had pores (6.6 ± 0.22) ÷ (10.0 ±0.32) μm in size (Table 1, Figure 1). Fibers had a smooth surface, and no pores above the lower resolution limit of SEM were detected (20–30 nm). As evident from SEM and AFM data, maximum roughness of 3D matrices was 4 μm. However, the average distance between the fibers located in a 0.5 μm thick surface layer was no more than 3 μm, thus providing a sufficient number of contacts with cellular adhesion molecules in a 200 μm^2^ area occupied by a single HUVEC (overall density of fibers was 43%, 35% and 30% for PCL, PCL-Gl, and PLGA, respectively). Previous reports of the effect of surface roughness on cell adhesion and proliferation are often conflicting and observed effects could instead be explained by the surface topography and chemical properties of the materials. For example, a study of HUVEC growth on polyurethanes and polyethylene-glycol modified polyurethanes with different degrees of nanoscale roughness showed that cells better adhere to and proliferate on the surfaces with 10–100 nm roughness rather than completely smooth surfaces [45]. Another study of HUVEC adhesion to electrospun 3D matrices and smooth films generated by a solvent casting method from poly(l-lactic acid) demonstrated that an increase of surface roughness from 135 nm to 1.5 µm decreased the efficacy of HUVEC adhesion and proliferation by 75%, but cells were still able to adhere to and proliferate on the surface of rough materials [8]. A recent report *showed rapid attachment and spreading of cells on 200 nm diameter PLGA mats* [46]. *In contrast, cell proliferation was dramatically increased for cells 600 nm and 1.5 μm diameter fibers after 14 days.* A number of sources demonstrated that, *for* sub-micron *fiber matrices, cell orientation and spreading, but not viability or proliferation can also be impacted by fiber alignment* [47,48,49]. The data on preferential adhesion and proliferation of HUVEC on a surface with hollows (5 × 5 µm with 5 µm long jumpers) than on a surface with both peaks (2 × 2 µm, 5 µm between centers) and hollows (5 × 5 µm, 5 µm apart) suggests that surface topology may be more important that roughness [50]. Thus, in case of fibrous materials, the roughness of the surface may not be the most critically important parameter for HUVEC adhesion and proliferation. Described reports as well as our own data suggest that if a thin surface layer (2–3 µm) of the material accommodates at least 2–3 fibers (0.1–1.5 µm) from cell-compatible polymer in a 10 × 10 µm area, such surface should enable HUVEC adhesion, though efficiency and speed of the process may vary.

Both PCL and PLGA are poorly wetted by water (contact angle 110–120° [51,52]), but the addition of Gl increased the hydrophilicity of the surface (Table 1) and was reported to promote better cell adhesion [53]. At that, hydrophobic surfaces can also be capable of binding cells [54,55], usually due to efficient protein binding [56]. Strength of PCL-based matrices varies from 1.8 to 2.5 MPa, depending on the composition of the ES solution [57,58], making them well suited for the production of small diameter VG. PLGA matrices are less elastic and compliant [59] but are frequently used in applications where biodegradation of the material is demanded.

### 3.2. Cultivation of HUVEC on 3D Matrices

**Figure 2:**
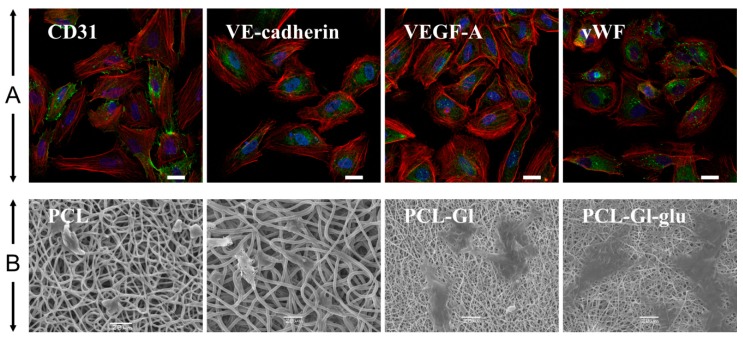
Characterization of HUVEC. (**A**) Immunofluorescence staining of HUVEC. Endothelial markers are stained green (AlexaFluor 488), F-actin and nuclei are stained red (Phalloidin-TRITC) and blue (Hoechst 33342), correspondingly. Scale bars represent 20 µm. (**B**) SEM images of HUVEC cells cultivated on the surface of matrices PCL, PLGA, PCL-Gl, and PCL-Gl-glu for two days.

**Figure 3:**
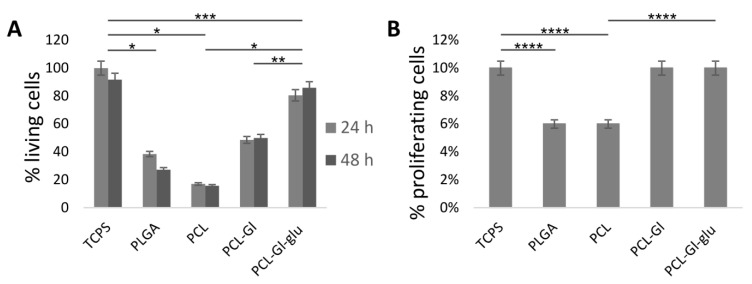
Cultivation of HUVEC on 3D matrices. (**A**) adhesion and proliferation of HUVEC cells (Alamar Blue) 24 and 48 h after seeding. Cells on 3D matrices are represented as % to cells on TCPS; (**B**) number of proliferating cells as evidenced by EdU assay. Bars indicate standard error of the mean. Statistically significant differences at * *p* < 0.001, ** *p* < 0.01, ***p = 0.05 (24 and 48 h); **** *p* < 0.05.

Endothelial status of HUVEC was confirmed by positive staining for endotheliocyte-specific antigens, including VEGF-A, VE-Cadherin, von Willebrand factor, and CD31 (PECAM1) (Figure 2). Doubling time of HUVEC grown on TCPS was ~36 h, while the same cells seeded on matrices exhibited variation within a ±15% range. This is close to the data obtained for Gl or poly-L-lactic acid films showing peak HUVEC density of 8–10 × 10^3^ cells/cm^2^ and doubling time between 24 h and 48 h depending on seeding density [60]. HUVEC, cultivated on PCL-GL matrices, were morphologically similar to cells grown in well plates or glass slides (Fig. 2). Cells better spread out on the surface of PCL-Gl-glu matrices, while the surface of PCL and PLGA matrices provided less favorable conditions for HUVEC adhesion. Cells seeded on these matrices had fewer contacts with the surface and could not spread out (Figure 2). It was found that, compared to TCPS, only 17% of HUVEC adhered to PCL matrices, 48% to PCL-Gl, and 80% to PCL-Gl-glu matrices (Figure 3A). PLGA matrices supported adhesion of ~38% of HUVEC. Cell proliferation efficacy calculated as a percentage of cells that incorporated EdU was similar for TCPS, PCL-Gl, and PCL-Gl-glu matrices, but significantly lower when cells were seeded on pure PCL or PLGA (Figure 3B).

According to these data, PCL-Gl-glu matrices provided the best substratum for adhesion and proliferation of HUVEC. This confirms our previous results demonstrating that modification of the surface with proteins recognizable by cellular receptors can increase cell adhesion [20]. The exposure of Gl on the surface of the matrices, especially when its mobility is limited by fixation with glutaraldehyde, can allow such materials to mimic the properties of natural biological membranes and promote efficient HUVEC adhesion and proliferation [44].

### 3.3. Transcriptome Profiling

RNA was isolated from cells cultured on electrospun matrices and TCPS. All RNA samples except PLGA_2 had RIN >6 (Table S1). A total of 129 million reads were obtained for 10 libraries on a HiSeq2500 platform using single-end 50 bp sequencing. Overall alignment rate was above 95% and a percentage of uniquely mapped reads was in a range of 82–90%. Thus, even a PLGA_2 library prepared from a low RIN RNA sample could be involved in the subsequent analysis. The good correlation between RNA samples from different biological donors grown in the same conditions is also notable (R = 0.896 ÷ 0.964), demonstrating high convergence of biological duplicates. 

To capture the changes in gene expression of different matrices, data from two donors were combined and differentially expressed (DE) genes were identified using DESeq2. Pairwise comparisons against HUVEC cultivated on TCPS, PCL or PCL-Gl-glu matrices are presented in Table 2. To potentially account for strength of the effects on phenotype, all genes were divided into categories based on RPKM: very low (0 < RPKM < 1), low (1 < RPKM< 10), average (10 < RPKM < 100), high (RPKM > 100), very high expression (RPKM > 1000), similar to previous studies [61,62] (Table 2 and Table 3). Genes expressed at a very low level (0 < RPKM < 1) were excluded from further analysis. 

Common characteristics of matrices should trigger the changes in the expression of same genes, resulting in similar sets of DE genes (common DE genes), from which genes involved in cellular response to different properties of 3D matrices can be identified (Table 2 and Table 3). Structure and chemical composition of the fibers presumably affect common DE genes in comparisons of electrospun matrices (PCL, PCL-Gl, PCL-Gl-glu, PLGA) against TCPS. As suggested previously [63], such common DE genes might be responsible for facilitating cell contacts with a fibrous matrix. Analysis of the comparisons between PCL-PLGA and PCL-PCL-Gl shows genes influenced by the chemical composition of the fibers (e.g., presence of gelatin), common DE genes in PCL-Gl-glu–PCL, and PCL-Gl-glu-PCL-Gl; comparisons should reveal genes affected by glutaraldehyde treatment.

**Figure 4:**
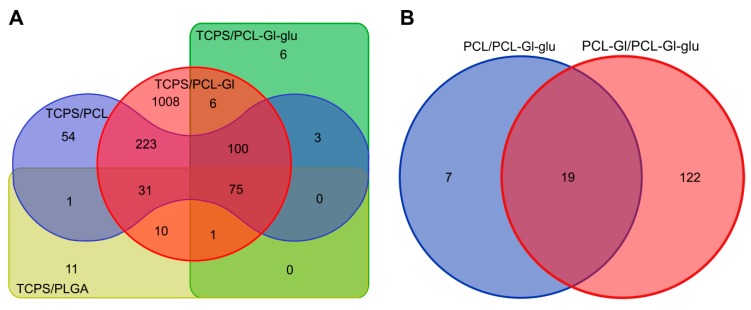
Venn diagrams of common DE genes. (**A**) comparisons of electrospun matrices with TCPS; (**B**) comparisons of PCL-based matrices.

HUVEC cultivated on all 3D matrices had significant DE genes compared to cells grown on TCPS (Figure 4). From 75 common DE genes for comparisons of electrospun 3D matrices with TCPS 7–9 upregulated genes (depending on the type of matrices) were in the average and only 1–3 in the high RPKM category (Table 2). Similarly, 11–20 genes were upregulated with FC 2–3 and 1–3 genes–with FC 3–5 (Table 3). On PCL-Gl-glu matrices, 21 genes were upregulated with FC 1–2 and just one with FC 2–3 (Table 3). Few genes in the average or high expression categories had FC 2–3. In cells grown on PCL-Gl, we found no upregulated genes with FC > 3 and no genes were deregulated with FC > 3 in cells cultivated on PCL-Gl-glu or PLGA matrices (Table S2). DE genes downregulated in cells cultivated on matrices were almost exclusively in the low or very low expression categories. 

PCL-Gl had the largest number of DE genes, including those in moderate, high, and very high expression categories (Table 2) and in all FC ranges (Table 3). The second highest number of DE genes in all ranges of RPKM and FC was between cells cultivated on PCL matrices and TCPS. HUVEC cultivated on PCL, PLGA, and PCL-Gl were very similar in terms of gene expression with no significant DE genes (Table 2). Cells from PLGA matrices most closely resembled cells grown on TCPS judging by the lowest number of DE genes. Cells from PCL-Gl-glu had 50% more DE genes compared to TCPS than those grown on PLGA matrices, but, in cells grown on PCL-Gl-glu, most genes were only slightly upregulated (22 with FC 1-2, 1 with FC 2-3), whereas on PLGA matrices the upregulation was somewhat higher numerically (18 with FC 1-2 and 11 with FC 2-3) (Table 3). In cells cultivated on PCL-Gl-glu matrices, the majority of DE genes were downregulated with FC less than 2. Based on these numbers and data from Figure 3, PCL-Gl-glu matrices can be considered as suitable counterparts to TCPS as far as HUVEC cultivation is concerned. 

PLGA 3D matrices are more hydrophilic than PCL (Table 1) and their ability to support HUVEC growth has been demonstrated earlier [64]. A lack of pronounced differences in gene expression agrees with data, showing that HUVEC better adhere and grow on PLGA matrices than on PCL, but worse than on PCL-Gl (Figure 2 and Figure 3). PCL-Gl-glu matrices differ from PCL-Gl only by treatment with glutaraldehyde, which leads to protein cross-linking and stabilization of the fiber’s surface structure. This suggests that the presence of mobile molecules on the surface not only decreases adhesion and proliferation of HUVEC (Figure 3), but can also cause changes in gene expression and alter cellular phenotype of adherent cells. Natural support for endothelial cells consists of insoluble elastin and collagen fibers of the basal membrane, which are assembled from soluble precursors but lose solubility as the fiber is formed [65]. Unsurprisingly, HUVEC seems to prefer a solid surface, which mimics their natural substrate. Materials that achieve facilitating their function result in the emergence of a normally functioning endothelium.

### 3.4. GO Enrichment and Functional Analysis of DE Genes

**Figure 5:**
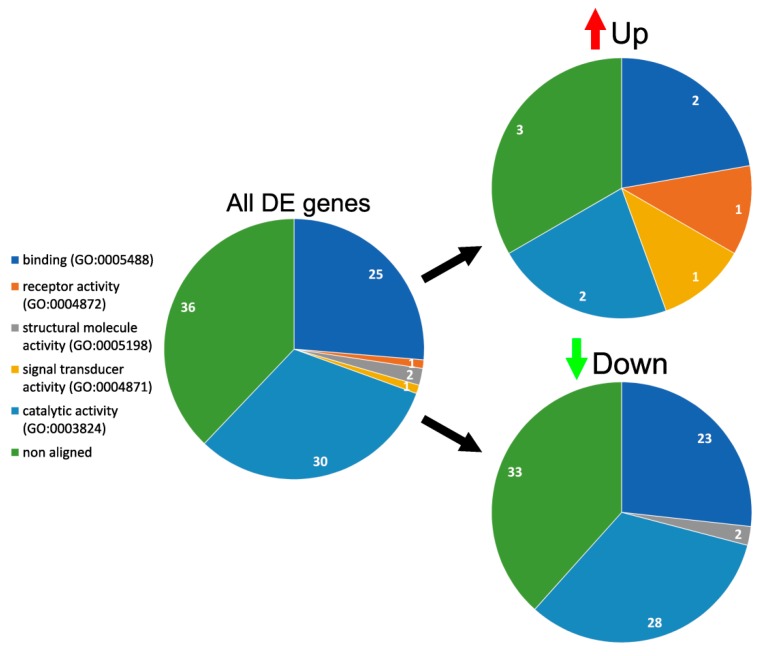
Molecular functions of DE genes in HUVEC cultivated on 3D matrices. Functions of DE genes expressed in HUVEC cultivated on matrices in comparison with TCPS according to the Gene Ontology Panther classification system. The number of genes in the group is depicted in each segment.

According to Gene Ontology (Panther classification system), common DE genes deregulated in cells on 3D matrices can be divided into five groups based on their molecular function (Figure 5). Upregulated genes were found in four groups: binding (two genes), catalytic activity (two genes), receptor activity (1), and signal transduction (1). Downregulated belonged in three groups: catalytic activity (28 genes), binding (23 genes), and structural molecule activity (two genes).

**Figure 6:**
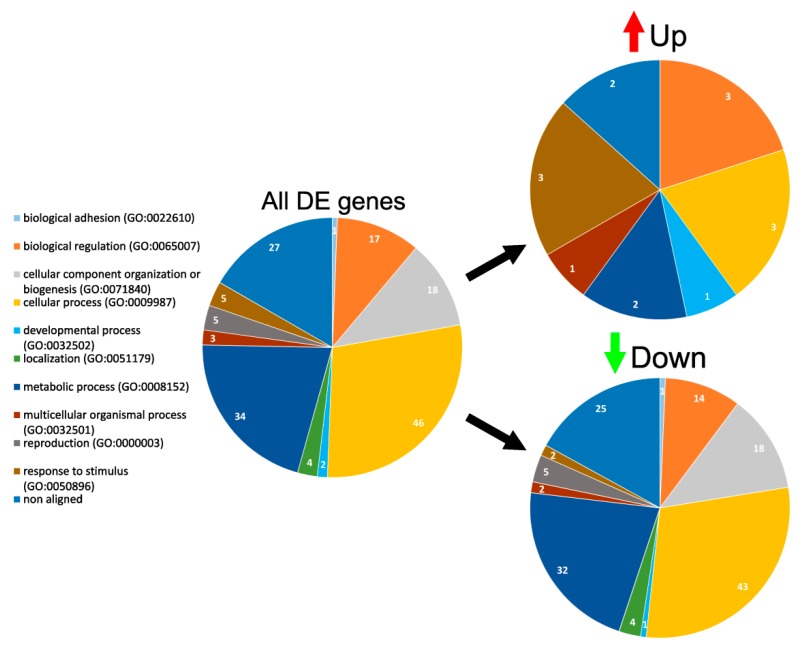
Biological processes of DE genes in HUVEC cultivated on 3D matrices. Biological processes related to DE genes expressed in HUVEC cultivated on matrices in comparison with TCPS according to the Gene Ontology Panther classification system. The number of genes in the group is depicted in each segment.

Biological processes encompassing these genes can be divided into 10 groups (Figure 6). Upregulated genes were found only in six groups including biological regulation (3), cellular processes (3), developmental processes (1), metabolic processes (2), multicellular organismal processes (one gene) and response to stimulus (3). Downregulated genes were present in all 10 groups: biological adhesion (1), biological regulation (14), cellular component regulation or biogenesis (18), cellular process (43), developmental process (1), localization (4), metabolic process (14) multicellular organismal process (2), reproduction (5), and response to stimulus (2). Downregulated genes were responsible for binding, catalytic, and metabolic activity (TYMS, MX1, MICAL2, AKAP12 for comparisons of TCPS with PCL, PCL-Gl, and PLGA, and GLIPR1 for TCPS and PLGA). Since few were expressed on levels above average and downregulation of such genes may suggest redundancy of their activity for cells cultivated on fibrillary matrices, downregulated genes were excluded from further analysis.

Several upregulated DE genes are of particular interest in the context of endothelization. HMOX1 was highly expressed in HUVEC cultivated on all 3D matrices compared to TCPS. Additionally, cells cultivated on PCL-Gl and PLGA expressed AQP1 and VIPR1 on an average level, while TSPAN7 and PTGR1 were expressed by HUVEC at an average level only on PCL-Gl. 

HMOX1 encodes for heme oxygenase 1 (HO-1)—a stress-inducible enzyme that catabolizes heme. HMOX1 is regulated at the transcriptional level, and can be induced by heme and various other agonists through signal transduction pathways associated with the production of reactive oxygen species by the mitochondria and/or the endoplasmic reticulum [66]. Active expression of HMOX1 could be a positive indicator of matrix endothelization since it is involved in vascular protection [67], cytoprotective effects against vascular injury, including post-transplant vasculopathy, allograft rejection, and ischemia reperfusion [68], suppression of endothelial cell apoptosis [69], expression of adhesion molecules [70], and salutary effects against immune-mediated inflammatory diseases [71]. Aquaporin 1 encoded by an AQP1 gene is highly expressed in most microvascular endothelial cells [72,73]. Ischemia, hypoxia, and cardioplegia reduce cardiac AQP1 level [74], while AQP1 knockdown results in high permeability of pulmonary microvasculature [75]. In contrast, AQP1 expression is required for hypoxia-inducible angiogenesis in human retinal vascular endothelial cells and endothelial cell migration [76,77]. This suggests that AQP1 expression can be beneficial for endothelialization of vascular grafts. Vasoactive Intestinal Peptide Receptor 1 encoded by VIPR1 gene also demonstrated a cytoprotective effect by inducing antiapoptotic signaling [78] and thus can be considered a positive factor of surface endothelialization. Functions of tetraspanin-cell-surface protein encoded by TSPAN7 gene include mediation of signal transduction events that play a role in the regulation of cell development, activation, growth, and motility [79]. Prostaglandin Reductase 1 (PTGR1) related pathways are prostaglandin and arachidonic acid metabolism associated with inflammation [80,81]. 

We have identified 100 common DE genes when comparing cells grown on different PCL-based matrices to TCPS (Table 2 and Table 3, Table S2). The surface composition of all these matrices is significantly different from TCPS and common DE genes identified in these comparisons could be associated with the response to the structure and chemical composition of the matrix surface. Only three DE genes were expressed at an average level in cells cultivated on PCL and PCL-Gl with reasonable FC (C1orf21, BMPER, DUSP5, FC > 2), and no such genes were found in cells from PCL-Gl-glu. At that, two genes expressed at very high level (FTH1, FTL) were upregulated (FC 1-2) in cells cultivated on all 3D matrices (Table S2). These genes are involved in iron storage in a soluble and nontoxic form, suggesting a possible connection with HMOX1 upregulation.

Similarly, DE genes in HUVEC cultivated on PCL-Gl-glu as compared to PCL and PCL-Gl matrices (Table 2 and Table 3) are supposedly affected by the interaction of cells with the fibers. As mentioned previously, PCL-Gl-glu are only different from PCL-Gl by the restricted mobility of Gl. HUVEC cultivated on PCL-Gl-glu differed from those cultivated on PCL and PCL-Gl by expression of 26 and 141 genes, respectively. Among those, 19 DE genes were shared in both comparisons, including three highly expressed genes (ANKRD1, CTGF, CYR61) upregulated in cells cultivated on PCL-Gl-glu matrices with FC < 2 (Table S2). These genes are responsible for endothelial cell proliferation, adhesion, migration, etc. [82,83], while five genes with average expression are involved in maintaining cell polarity and signal transduction (CLDN11) [84,85], proliferation (DKK) [86], development and cell-to-cell signaling (NRG1) [87,88], and immune response (IL6 and IL1RL1) [89]. Upregulation of these genes in cells actively proliferating on PCL-Gl-glu is plausible. Out of seven genes deregulated in PCL-Gl-glu but not PCL-Gl as compared to PCL 6 were mildly downregulated (FC < 1,4). Late endosomal protein LAMP3 [90] was slightly upregulated FC = 1,24, but its expression was below 1.5 RPKM for both matrix types. Gene expression profiles of HUVEC cultivated on PCL-Gl-glu were closer to PCL than PCL-Gl matrices, despite the data demonstrating more efficient adhesion and proliferation of HUVEC on PCL-Gl-glu (Figure 3). These data suggest significant contributions of not only chemical composition but also mobility of the surface layer to the efficiency of cell-to-matrix interactions.

Thus, both functional testing and gene expression profiling suggest PCL-Gl-glu matrices as a suitable surface for HUVEC adhesion and proliferation. Gene expression profiling is a robust tool that can reveal cause-and-effect relationships between cellular phenotype and principal features of the materials, such as surface structure and chemical composition of 3D matrices, and indicate expression markers of specific cell properties. Here, we detected activation of genes mediating desirable cellular functions, namely, adhesion, proliferation, inhibition of ectopic tissue calcification and inflammation, etc. One caveat is that comparison of the cells cultivated on electrospun materials and TCPS is not entirely valid. TCPS is a smooth, non-permeable artificial support that does not enable many of the endothelial cell functions—barrier, transport, etc. Moreover, in vivo endothelial cells are influenced by shearing forces [91,92] and mechanical deformation of the support [93,94], which differ depending on the localization of vessel in the body and determine their utility for testing of the endothelization of the materials. 

## 4. Conclusions

In summary, 3D matrices fabricated by electrospinning from solutions of PLGA, PCL, and PCL blends with Gl were studied as substrates for endothelization by HUVECPCL-Gl-glu matrices were found to be suitable for cultivation of functional HUVEC cells and production of the inner layer of vascular grafts. Transcriptome sequencing demonstrated a minimal difference of the gene expression profile in HUVEC cultivated on PCL-Gl-glu matrices compared to TCPS, confirming these matrices as the best support for endothelization.

## Figures and Tables

**Table 1:** Electrospinning conditions and properties of fabricated 3D matrices.

Blend Composition	Electrospinning Parameters			Characteristics of Matrices		
	Voltage, kV	Solution Supply Rate, mL/h	Capillary-collector Distance, cm	Fiber Diameter, µm	Pore Size, µm	Contact Angle, °
PCL	23.5	1.5	20	1.05 ± 0.12 ^1^	6.6 ± 0.22 ^1^	127.5 ± 1.29 ^2^
PCL-Gl	24.5	1.3	20	0.89 ± 0.09	7.0 ± 0.57	85.66 ± 2.9
PLGA	20.0	1.5	20	1.12 ± 0.13	10.0 ± 0.32	113.5 ± 1.3

^1^ mean ± standard error of the mean. ^2^ mean ± standard deviation.

**Table 2:** Differentially expressed (DE) genes and common DE genes for comparison groups.

No.	Control	Comparison	DE Genes					
			Total	by RPKM1	Common		Common, by RPKM ^1^	
1	TCPS	PCL	487	152/167/140/24/4	100	75	42/41/15/0/2	46/19/8/2/0
2		PCL-Gl	1454	268/493/478/176/39			45/37/15/1/2	47/18/8/2/0
3		PCL-Gl-glu	191	47/106/31/5/2			27/54/17/0/2	17/48/7/3/0
4		PLGA	129	58/35/29/6/1				46/19/9/1/0
5	PCL-Gl-glu	PCL	26	5/6/10/4/1	19		4/6/5/2/1	
6		PCL-Gl	141	37/37/44/21/2			4/6/5/2/1	
7	PCL	PLGA	0	-	-		-	
8		PCL-Gl	0	-			-	

^1^ RPKM values shown as counts in the intervals 0 < RPKM < 1/1 ≤ RPKM < 10/10 ≤ RPKM < 100/100 ≤ RPKM < 1000 and RPKM > 1000.

**Table 3:** Distribution of differentially expressed (DE) genes by fold change. Total number of DE genes and number of common DE genes in different comparison groups are shown.

No.	Control	Comparison	DE Genes, Up/Down							
			Total	Common		by FC				
						1–2	2–3	3–4	4–5	>5
**1**	TCPS	PCL	125/362	100	75	112/219 [7/35] ^1^ (5/3) ^2^	12/86 [3/42] (0/29)	1/43 [0/11)] (1/29)	0/13 [0/2] (0/8)	0/1 [0/0] (0/0)
**2**		PCL-Gl	657/797			635/602 [7/31] (1/1)	20/105 [3/32] (4/18)	2/58 [0/22] (1/30)	0/23 [0/5] (0/16)	0/9 [0/0] (0/4)
**3**		PCL-GL-glu	22/169			21/159 [10/88] (5/62)	1/10 [0/2] (1/7)	0/0 [0/0] (0/0)	0/0 [0/0] (0/0)	0/0 [0/0] (0/0)
**4**		PLGA	30/99			18/17 (3/6)	11/61 (3/49)	0/20 (0/14)	1/1 (0/0)	0/0 (0/0)
**5**	PCL-Gl-glu	PCl	13/13	19		13/13 (7/12)	0/0 (0/0)	0/0 (0/0)	0/0 (0/0)	0/0 (0/0)
**6**		PCL-Gl	47/96			46/93 (7/11)	1/1 (1/0)	0/0 (0/0)	0/0 (0/0)	0/0 (0/0)

^1^ Common DE genes for comparisons 1–3 are given in square brackets. ^2^ Common DE genes for comparisons 1–4 and 5–6 are given in round brackets.

## Supplementary Materials

The following are available online at https://www.mdpi.com/1996-1944/12/24/4082/s1, Table S1: RNA quality, sequencing and alignment summary, Table S2: Fold change (FC) distribution of common DE genes by expression level.

## References

[1] Liu T., Liu S., Zhang K., Chen J., Huang N. (2014). Endothelialization of implanted cardiovascular biomaterial surfaces: The development from in vitro to in vivo. J. Biomed. Mater. Res. Part A.

[2] Seifalian A.M., Tiwari A., Hamilton G., Salacinski J.H. (2002). Improving the Clinical Patency of Prosthetic Vascular and Coronary Bypass Grafts: The Role of Seeding and Tissue Engineering. Artif. Organs.

[3] Aubin H., Mas-Moruno C., Iijima M., Schutterle N., Steinbrink M., Assmann A., Gil F.J., Lichtenberg A., Pegueroles M., Akhyari P. (2016). Customized Interface Biofunctionalization of Decellularized Extracellular Matrix: Toward Enhanced Endothelialization. Tissue Eng. Part C Methods.

[4] Li S., Henry J.J.D. (2011). Nonthrombogenic Approaches to Cardiovascular Bioengineering. Annu. Rev. Biomed. Eng..

[5] Natorska J., Undas A. (2015). Blood coagulation and fibrinolysis in aortic valve stenosis: Links with inflammation and calcification. Thromb. Haemost..

[6] Chistiakov D.A., Orekhov A.N., Bobryshev Y.V. (2017). Effects of shear stress on endothelial cells: Go with the flow. Acta Physiol..

[7] Collins C., Osborne L.D., Guilluy C., Chen Z., O’Brien E.T., Reader J.S., Burridge K., Superfine R., Tzima E. (2014). Haemodynamic and extracellular matrix cues regulate the mechanical phenotype and stiffness of aortic endothelial cells. Nat. Commun..

[8] Xu C., Yang F., Wang S., Ramakrishna S. (2004). In vitro study of human vascular endothelial cell function on materials with various surface roughness. J. Biomed. Mater. Res..

[9] Montoya Y., Valencia R.A., Ortiz I.C., Hoyos L.M., Bustamante J. (2017). In Vitro Study of Proliferation and Cellularisation on Electrospun Membranes for Vascular Prosthesis. VII Latin American Congress on Biomedical Engineering CLAIB 2016, Bucaramanga, Santander, Colombia, October 26th–28th, 2016.

[10] Pashneh-Tala S., MacNeil S., Claeyssens F. (2016). The Tissue-Engineered Vascular Graft—Past, Present, and Future, Tissue Eng. Part B Rev..

[11] Manea L.R., Hristian L., Leon A.L., Popa A. (2016). Recent Advances of Basic Materials to Obtain Electrospun Polymeric Nanofibers for Medical Applications. IOP Conference Series: Materials Science and Engineering.

[12] Stepanova A.O., Chernonosova V.S., Popova I.V., Karpenko A.A., Pokushalov E.A., Karaskov A.M., Laktionov P.P., Vlassov V.V. (2016). Method for Producing Small-Diameter Low-Porosity Vascular Prostheses (Versions).

[13] Henry J.D., Yu J., Wang A., Lee R., Fang J., Li S. (2017). Engineering the mechanical and biological properties of nanofibrous vascular grafts for in situ vascular tissue engineering. Biofabrication.

[14] Catto V., Fare S., Cattaneo I., Figliuzzi M., Alessandrino A., Freddi G., Remuzzi A., Tanzi M.C. (2015). Small diameter electrospun silk fibroin vascular grafts: Mechanical properties, in vitro biodegradability, and in vivo biocompatibility. Mater. Sci. Eng. C.

[15] Nicast Nano-fibrous Medical Devices. http://nicast.com/.

[16] D’Amore A., Luketich S.K., Raffa G.M., Olia S., Menallo G., Mazzola A., D’Accardi F., Grunberg T., Gu X., Pilato M. (2018). Heart valve scaffold fabrication: Bioinspired control of macro-scale morphology, mechanics and micro-structure. Biomaterials.

[17] Gong W., Lei D., Li S., Huang P., Qi Q., Sun Y., Zhang Y., Wang Z., You Z., Ye X. (2016). Hybrid small-Diameter vascular grafts: Anti-Expansion effect of electrospun poly ε-Caprolactone on heparin-Coated decellularized matrices. Biomaterials.

[18] Wang Z., Cui Y., Wang J., Yang X., Wu Y., Wang K., Gao X., Li D., Li Y., Zheng X.L. (2014). The effect of thick fibers and large pores of electrospun poly(ε-Caprolactone) vascular grafts on macrophage polarization and arterial regeneration. Biomaterials.

[19] Fu W., Liu Z., Feng B., Hu R., He X., Wang H., Yin M., Huang H., Zhang H., Wang W. (2014). Electrospun gelatin/PCL and collagen/PLCL scaffolds for vascular tissue engineering. Int. J. Nanomed..

[20] Chernonosova V.S., Kvon R.I., Stepanova A.O., Larichev Y.V., Karpenko A.A., Chelobanov B.P., Kiseleva E.V., Laktionov P.P. (2017). Human serum albumin in electrospun PCL fibers: Structure, release, and exposure on fiber surface. Polym. Adv. Technol..

[21] Olmer R., Engels L., Usman A., Menke S., Malik M.N.H., Pessler F., Göhring G., Bornhorst D., Bolten S., Abdelilah-Seyfried S. (2018). Differentiation of Human Pluripotent Stem Cells into Functional Endothelial Cells in Scalable Suspension Culture. Stem Cell Rep..

[22] Hauser S., Jung F., Pietzsch J. (2017). Human Endothelial Cell Models in Biomaterial Research. Trends Biotechnol..

[23] Munoz-Pinto D.J., Erndt-Marino J.D., Becerra-Bayona S.M., Guiza-Arguello V.R., Samavedi S., Malmut S., Reichert W.M., Russell B., Höök M., Hahn M.S. (2017). Evaluation of late outgrowth endothelial progenitor cell and umbilical vein endothelial cell responses to thromboresistant collagen-mimetic hydrogels. J. Biomed. Mater. Res. Part A.

[24] Kang T.Y., Lee J.H., Kim B.J., Kang J.A., Hong J.M., Kim B.S., Cha H.J., Rhie J.W., Cho D.W. (2015). In vivo endothelization of tubular vascular grafts through in situ recruitment of endothelial and endothelial progenitor cells by RGD-fused mussel adhesive proteins. Biofabrication.

[25] Ritarwan K., Lelo A., Pane Y.S., Nerdy N. (2018). Increasing Atherosclerosis in Streptozotocin-Induced Diabetes into Four Groups of Mice, Open Access Maced. J. Med. Sci..

[26] Bouis D., Hospers G.A.P., Meijer C., Molema G., Mulder N.H. (2001). Endothelium in vitro: A review of human vascular endothelial cell lines for blood vessel-Related research. Angiogenesis.

[27] Chennazhy K.P., Krishnan L.K. (2005). Effect of passage number and matrix characteristics on differentiation of endothelial cells cultured for tissue engineering. Biomaterials.

[28] Kievit F.M., Florczyk S.J., Leung M.C., Wang K., Wu J.D., Silber J.R., Ellenbogen R.G., Lee J.S.H., Zhang M. (2014). Proliferation and enrichment of CD133+ glioblastoma cancer stem cells on 3D chitosan-Alginate scaffolds. Biomaterials.

[29] Kargozar S., Mozafari M., Hashemian S.J., Milan P.B., Hamzehlou S., Soleimani M., Joghataei M.T., Gholipourmalekabadi M., Korourian A., Mousavizadeh K. (2018). Osteogenic potential of stem cells-Seeded bioactive nanocomposite scaffolds: A comparative study between human mesenchymal stem cells derived from bone, umbilical cord Wharton’s jelly, and adipose tissue. J. Biomed. Mater. Res. Part B Appl. Biomater..

[30] Ye K., Felimban R., Traianedes K., Moulton S.E., Wallace G.G., Chung J., Quigley A., Choong P.F.M., Myers D.E. (2014). Chondrogenesis of Infrapatellar Fat Pad Derived Adipose Stem Cells in 3D Printed Chitosan Scaffold. PLoS ONE.

[31] Pekar-Zlotin M., Hirsch F.R., Soussan-Gutman L., Ilouze M., Dvir A., Boyle T., Wynes M., Miller V.A., Lipson D., Palmer G.A. (2015). Fluorescence In Situ Hybridization, Immunohistochemistry, and Next-Generation Sequencing for Detection of EML4-ALK Rearrangement in Lung Cancer. Oncologist.

[32] Khodakov D., Wang C., Zhang D.Y. (2016). Diagnostics based on nucleic acid sequence variant profiling: PCR, hybridization, and NGS approaches. Adv. Drug Deliv. Rev..

[33] Zhao L., Lu Y.T., Li F., Wu K., Hou S., Yu J., Shen Q., Wu D., Song M., OuYang W.H. (2013). High-Purity Prostate Circulating Tumor Cell Isolation by a Polymer Nanofiber-Embedded Microchip for Whole Exome Sequencing. Adv. Mater..

[34] Zhang G., Chen L., Guo X., Wang H., Chen W., Wu G., Gu B., Miao W., Kong J., Jin X. (2018). Comparative Analysis of microRNA Expression Profiles of Exosomes Derived from Normal and Hypoxic Preconditioning Human Neural Stem Cells by Next Generation Sequencing. J. Biomed. Nanotechnol..

[35] Eissa A.M., Barros F.S.V., Vrljicak P., Brosens J.J., Cameron N.R. (2018). Enhanced Differentiation Potential of Primary Human Endometrial Cells Cultured on 3D Scaffolds. Biomacromolecules.

[36] Hrdlickova R., Toloue M., Tian B. (2017). RNA-Seq methods for transcriptome analysis. Wiley Interdiscip. Rev. RNA.

[37] Jaffe E.A., Nachman R.L., Becker C.G., Miinick C.R. (1973). Culture of Human Endothelial Cells Derived from Umbilical Veins. J. Clin. Investig..

[38] Mal’shakova V.S., Laktionov P.P., Pyshnyi D.V., Vlasov V.V. (2007). Intracellular localization of natural and modified oligonucleotides in primary human endothelial cells. Bull. Exp. Biol. Med..

[39] Hannon G.J. (2010). FASTX-Toolkit. http://hannonlab.cshl.edu/fastx_toolkit.

[40] Kim D., Pertea G., Trapnell C., Pimentel H., Kelley R., Salzberg S.L. (2013). TopHat2: Accurate alignment of transcriptomes in the presence of insertions, deletions and gene fusions. Genome Biol..

[41] Liao Y., Smyth G.K., Shi W. (2014). Featurecounts: An efficient general purpose program for assigning sequence reads to genomic features. Bioinformatics.

[42] Love M.I., Huber W., Anders S. (2014). Moderated estimation of fold change and dispersion for RNA-Seq data with DESeq2. Genome Biol..

[43] Trapnell C., Williams B.A., Pertea G., Mortazavi A., Kwan G., Van Baren M.J., Salzberg S.L., Wold B.J., Pachter L. (2010). Transcript assembly and quantification by RNA-Seq reveals unannotated transcripts and isoform switching during cell differentiation. Nat. Biotechnol..

[44] Stepanova A.O., Chernonosova V.S., Kvon R.I., Laktionov P.P. Gelatin and Heparin Exposure on the Surface of Electrospun PCL Fibers. Proceedings of the TERMIS-EU 2016 Conference.

[45] Chung T.W., Liu D.Z., Wang S.Y., Wang S.S. (2003). Enhancement of the growth of human endothelial cells by surface roughness at nanometer scale. Biomaterials.

[46] Ko Y.G., Park J.H., Lee J.B., Oh H.H., Park W.H., Cho D., Kwon O.H. (2016). Growth behavior of endothelial cells according to electrospun poly(D,L-Lactic-Co-Glycolic acid) fiber diameter as a tissue engineering scaffold. Tissue Eng. Regen. Med..

[47] Whited B.M., Rylander M.N. (2014). The influence of electrospun scaffold topography on endothelial cell morphology, alignment, and adhesion in response to fluid flow. Biotechnol. Bioeng..

[48] Ahmed M., Ramos T., Wieringa P., Van Blitterswijk C., De Boer J., Moroni L. (2018). Geometric constraints of endothelial cell migration on electrospun fibres. Sci. Rep..

[49] Bashur C.A., Dahlgren L.A., Goldstein A.S. (2006). Effect of fiber diameter and orientation on fibroblast morphology and proliferation on electrospun poly(D,L-Lactic-Co-Glycolic acid) meshes. Biomaterials.

[50] Lutter C., Nothhaft M., Rzany A., Garlichs C.D., Cicha I. (2015). Effect of specific surface microstructures on substrate endothelialisation and thrombogenicity: Importance for stent design. Clin. Hemorheol. Microcirc..

[51] Ghasemi-Mobarakeh L., Prabhakaran M.P., Morshed M., Nasr-Esfahani M.H., Ramakrishna S. (2008). Electrospun poly(ɛ-Caprolactone)/gelatin nanofibrous scaffolds for nerve tissue engineering. Biomaterials.

[52] Zhang Y., Ouyang H., Lim C.T., Ramakrishna S., Huang Z.M. (2005). Electrospinning of gelatin fibers and gelatin/PCL composite fibrous scaffolds. J. Biomed. Mater. Res..

[53] Jiang Y.C., Jiang L., Huang A., Wang X.F., Li Q., Turng L.S. (2017). Electrospun polycaprolactone/gelatin composites with enhanced cell–Matrix interactions as blood vessel endothelial layer scaffolds. Mater. Sci. Eng. C..

[54] Saltzman W.M., Kyriakides T.R. (2014). Cell Interactions with Polymers. Principles of Tissue Engineering.

[55] Oliveira S.M., Alves N.M., Mano J.F. (2014). Cell interactions with superhydrophilic and superhydrophobic surfaces. J. Adhes. Sci. Technol..

[56] Mohan T., Niegelhell K., Nagaraj C., Reishofer D., Spirk S., Olschewski A., Kleinschek K.S., Kargl R. (2017). Interaction of Tissue Engineering Substrates with Serum Proteins and Its Influence on Human Primary Endothelial Cells. Biomacromolecules.

[57] Stepanova A.O., Korobeinikov M.V., Yunoshev A.S., Laktionov P.P. (2015). Effect of Electron-Beam Irradiation on Electrospinning Produced Scaffolds. 2015 International Conference on Biomedical Engineering and Computational Technologies.

[58] Hiep N.T., Lee B.T. (2010). Electro-Spinning of PLGA/PCL blends for tissue engineering and their biocompatibility. J. Mater. Sci. Mater. Med..

[59] Baker S.C., Rohman G., Southgate J., Cameron N.R. (2009). The relationship between the mechanical properties and cell behaviour on PLGA and PCL scaffolds for bladder tissue engineering. Biomaterials.

[60] Heng B.C., Bezerra P.P., Preiser P.R., Law S.K.A., Xia Y., Boey F., Venkatraman S.S. (2011). Effect of cell-Seeding density on the proliferation and gene expression profile of human umbilical vein endothelial cells within ex vivo culture. Cytotherapy.

[61] Ding Z., Zhang Z., Zhong J., Luo D., Zhou J., Yang J., Xiao L., Shu D., Tan H. (2016). Comparative transcriptome analysis between an evolved abscisic acid-Overproducing mutant Botrytis cinerea TBC-A and its ancestral strain Botrytis cinerea TBC-6. Sci. Rep..

[62] Bhargava V., Head S.R., Ordoukhanian P., Mercola M., Subramaniam S. (2015). Technical Variations in Low-Input RNA-Seq Methodologies. Sci. Rep..

[63] Lother A., Bergemann S., Deng L., Moser M., Bode C., Hein L. (2018). Cardiac Endothelial Cell Transcriptome. Arterioscler. Thromb. Vasc. Biol..

[64] Seo H.J., Yu S.M., Lee S.H., Choi J.B., Park J.C., Kim J.K. (2009). Effect of PLGA Nano-Fiber/Film Composite on HUVECs for Vascular Graft Scaffold. 13th International Conference on Biomedical Engineering.

[65] Chi J.T., Chang H.Y., Haraldsen G., Jahnsen F.L., Troyanskaya O.G., Chang D.S., Wang Z., Rockson S.G., van de Rijn M., Botstein D. (2003). Endothelial cell diversity revealed by global expression profiling. Proc. Natl. Acad. Sci. USA.

[66] Gozzelino R., Jeney V., Soares M.P. (2010). Mechanisms of Cell Protection by Heme Oxygenase-1. Ann. Rev. Pharmacol. Toxicol..

[67] He C., Zhao C., Kumar A., Lee C., Chen M., Huang L., Wang J., Ren X., Jiang Y., Chen W. (2014). Vasoprotective effect of PDGF-CC mediated by HMOX1 rescues retinal degeneration. Proc. Natl. Acad. Sci. USA.

[68] Kinderlerer A.R., Gregoire I.P., Hamdulay S.S., Ali F., Steinberg R., Silva G., Ali N., Wang B., Haskard D.O., Soares M.P. (2008). Heme oxygenase-1 expression enhances vascular endothelial resistance to complement-Mediated injury through induction of decay-Accelerating factor: A role for increased bilirubin and ferritin. Blood.

[69] Brouard S., Otterbein L.E., Anrather J., Tobiasch E., Bach F.H., Choi A.M.K., Soares M.P. (2000). Carbon Monoxide Generated by Heme Oxygenase 1 Suppresses Endothelial Cell Apoptosis. J. Exp. Med..

[70] Seldon M.P., Silva G., Pejanovic N., Larsen R., Gregoire I.P., Filipe J., Anrather J., Soares M.P. (2007). Heme Oxygenase-1 Inhibits the Expression of Adhesion Molecules Associated with Endothelial Cell Activation via Inhibition of NF-B RelA Phosphorylation at Serine 276. J. Immunol..

[71] Vijayan V., Wagener F.A.D.T.G., Immenschuh S. (2018). The macrophage heme-Heme oxygenase-1 system and its role in inflammation. Biochem. Pharmacol..

[72] Au C.G., Cooper S.T., Lo H.P., Compton A.G., Yang N., Wintour E.M., North K.N., Winlaw D.S. (2004). Expression of aquaporin 1 in human cardiac and skeletal muscle. J. Mol. Cell. Cardiol..

[73] Kim J., Jung Y. (2011). Different expressions of AQP1, AQP4, eNOS, and VEGF proteins in ischemic versus non-Ischemic cerebropathy in rats: Potential roles of AQP1 and eNOS in hydrocephalic and vasogenic edema formation. Anat. Cell Biol..

[74] Bondy C., Chin E., Smith B.L., Preston G.M., Agre P. (1993). Developmental gene expression and tissue distribution of the CHIP28 water-Channel protein. Proc. Natl. Acad. Sci. USA.

[75] Rutkovskiy A., Bliksøen M., Hillestad V., Amin M., Czibik G., Valen G., Vaage J., Amiry-Moghaddam M., Stensløkken K.O. (2013). Aquaporin-1 in cardiac endothelial cells is downregulated in ischemia, hypoxia and cardioplegia. J. Mol. Cell. Cardiol..

[76] Kaneko K., Yagui K., Tanaka A., Yoshihara K., Ishikawa K., Takahashi K., Bujo H., Sakurai K., Saito Y. (2008). Aquaporin 1 is required for hypoxia-Inducible angiogenesis in human retinal vascular endothelial cells. Microvasc. Res..

[77] Papadopoulos M.C., Saadoun S., Verkman A.S. (2008). Aquaporins and cell migration. Pflug. Arch. Eur. J. Physiol..

[78] Sastry K.S., Chouchane A.I., Wang E., Kulik G., Marincola F.M., Chouchane L. (2017). Cytoprotective effect of neuropeptides on cancer stem cells: Vasoactive intestinal peptide-Induced antiapoptotic signaling. Cell Death Dis..

[79] (2019). RefSeq Gene: TSPAN7, NC_000023.11. https://www.ncbi.nlm.nih.gov/gene/7102.

[80] Vitturi D.A., Chen C.S., Woodcock S.R., Salvatore S.R., Bonacci G., Koenitzer J.R., Stewart N.A., Wakabayashi N., Kensler T.W., Freeman B.A. (2013). Modulation of Nitro-fatty Acid Signaling PROSTAGLANDIN REDUCTASE-1 IS A NITROALKENE REDUCTASE. J. Biol. Chem..

[81] Chou W.L., Chuang L.M., Chou C.C., Wang A.H.J., Lawson J.A., FitzGerald G.A., Chang Z.F. (2007). Identification of a Novel Prostaglandin Reductase Reveals the Involvement of Prostaglandin E 2 Catabolism in Regulation of Peroxisome Proliferator-Activated Receptor γ Activation. J. Biol. Chem..

[82] Samaras S.E., Chen B., Koch S.R., Sawyer D.B., Lim C.C., Davidson J.M. (2012). 26S Proteasome regulation of Ankrd1/CARP in adult rat ventricular myocytes and human microvascular endothelial cells. Biochem. Biophys. Res. Commun..

[83] Brigstock D.R. (2002). Regulation of angiogenesis and endothelial cell function by connective tissue growth factor (CTGF) and cysteine-Rich 61 (CYR61). Angiogenesis.

[84] Karagiannis G.S., Schaeffer D.F., Cho C.K.J., Musrap N., Saraon P., Batruch I., Grin A., Mitrovic B., Kirsch R., Riddell R.H. (2014). Collective migration of cancer-Associated fibroblasts is enhanced by overexpression of tight junction-Associated proteins claudin-11 and occluding. Mol. Oncol..

[85] Findley M.K., Koval M. (2009). Regulation and roles for claudin-Family tight junction proteins. Iubmb Life..

[86] Huang Y., Liu L., Liu A. (2018). Dickkopf-1: Current knowledge and related diseases. Life Sci..

[87] Johns E.J. (2014). Neuregulin and the ErbB signalling cascade in cardiovascular control. J. Hypertens..

[88] Britsch S. (2007). The neuregulin-I/ErbB signaling system in development and disease. Adv. Anat. Embryol. Cell Biol..

[89] Tanaka T., Narazaki M., Kishimoto T. (2014). IL-6 in Inflammation, Immunity, and Disease. Cold Spring Harb. Perspect. Biol..

[90] Kobayashi T., Vischer U.M., Rosnoblet C., Lebrand C., Lindsay M., Parton R.G., Kruithof E.K.O., Gruenberg J. (2000). The Tetraspanin CD63/lamp3 Cycles between Endocytic and Secretory Compartments in Human Endothelial Cells. Mol. Biol. Cell..

[91] Fisher A.B., Chien S., Barakat A.I., Nerem R.M. (2001). Endothelial cellular response to altered shear stress. Am. J. Physiol. Cell. Mol. Physiol..

[92] Li Y.S.J., Haga J.H., Chien S. (2005). Molecular basis of the effects of shear stress on vascular endothelial cells. J. Biomech..

[93] Wang J.H., Goldschmidt-Clermont P., Wille J., Yin F.C. (2001). Specificity of endothelial cell reorientation in response to cyclic mechanical stretching. J. Biomech..

[94] Brown T.D. (2000). Techniques for mechanical stimulation of cells in vitro: A review. J. Biomech..

