# Supplementary material for: General Study and Gene Expression Profiling of Endotheliocytes Cultivated on Electrospun Materials
Source: Materials (Basel). 2019 Dec 6;12(24):4082. doi: 10.3390/ma12244082 (PMC6947544; doi:10.3390/ma12244082)
Supplement: Supplementary file 1 [file materials-12-04082-s001.pdf]

materials

MDPI

Supplementary Materials

General Study and Gene Expression Profiling of Endotheliocytes Cultivated on Electrospun Materials

Alena O. Stepanova [1,2,*], Petr P. Laktionov [3,4], Anna V. Cherepanova [1,2], Vera S. Chernonosova [1,2], Georgiy Yu. Shevelev [5], Ivan A. Zaporozhchenko [1,2], Alexander M. Karaskov [1] and Pavel P. Laktionov [1,2]

[1] Laboratory of Biomedical Technologies, Meshalkin National Medical Research Center, Ministry of Health of the Russian Federation, Rechkunovskaya str. 15, 630055 Novosibirsk, Russia; a_cher@niboch.nsc.ru (A.V.C.), vera_mal@niboch.nsc.ru (V.S.C.), ivanzap@niboch.nsc.ru (I.A.Z.), meshalkin@meshalkin.ru (A.M.K.), lakt@niboch.nsc.ru (P.P.L.)

[2] Laboratory of Molecular Medicine, Institute of Chemical Biology and Fundamental Medicine, Siberian Branch of the Russian Academy of Sciences (ICBFM SB RAS), Lavrentiev ave. 8, 630090 Novosibirsk, Russia.

[3] Department of the Regulation of Genetic Processes, Laboratory of Genomics, Institute of Molecular and Cell Biology, Siberian Branch of the Russian Academy of Sciences (IMCB SB RAS), Lavrentiev ave. 8/2, 630090 Novosibirsk, Russia; laktionov@mcb.nsc.ru

[4] Department of Natural Sciences, Epigenetics Laboratory, Novosibirsk State University, Pirogova str. 2, 630090 Novosibirsk, Russia.

[5] Laboratory of Biomedical Chemistry, Institute of Chemical Biology and Fundamental Medicine, Siberian Branch of the Russian Academy of Sciences (ICBFM SB RAS), Lavrentiev ave. 8, 630090 Novosibirsk, Russia; shevelev@niboch.nsc.ru

* Correspondence: lebedeva@niboch.nsc.ru; Tel.: +7-(383)-363-51-44

Received: 24 October 2019; Accepted: 3 December 2019; Published: date

Table S1. RNA quality, sequencing and alignment summary.

Library id	RNA Quality, RIN/RQN†	Total Number of Reads	Uniquely Mapped Reads	Reads Mapping to Multiple Locations	Reads Mapping to Human Genome, %	Uniquely Mapped Reads Vs Total, %	Correlation, r
Contr_1 (TCPS)	7.9/9.9	15474341	13898163	1526499	96.8	89.8	0.974
Contr_2 (TCPS)	7.1/9.5	11572847	10377990	1155328	96.7	89.7	
PCL_1	7.4/7.3	15193394	13266763	1850704	96.1	87.3	0.911
PCL_2	6.9/7.0	13078913	11161978	1854031	96.7	85.3	
PCL-Gl_1	7.4/7.0	14795234	12163491	2553152	97.0	82.2	0.941
PCL-Gl_2	6.6/7.6	12710767	10613545	2031721	96.9	83.5	
PCL-Gl-glu_1	7.8/8.9	13458206	11933017	1477035	96.8	88.7	0.848
PCL-Gl-glu_2	6.8/7.6	13031393	11357181	1621520	96.9	87.2	
PLGA_1	7.4/7.0	5812354	4969853	698263	82.9	85.5	0.760
PLGA_2	5.9/6.0	13840171	12154880	1632403	96.9	87.8	

† RNA quality was assessed with Advanced Analytical Fragment Analyzer or Agilent Bioanalyzer 2100.

Table S2. Fold change (FC) distribution of common DE genes by expression level.

Common DE Genes	Control	Comparison	RPKM	DE Genes, Up/Down				
				FC				
				1–2	2–3	3–4	4–5	>5
75	TCPS	PCL	0–1		0/13	0/26	0/7	
			Low 1–10	0/1	0/14	0/3	0/1	
			Middle 10–100	4/2	0/2			
			High 100–1000	1/0		1/0		
			Highest > 1000					
		PCL-Gl	0–1		0/4	0/24	0/15	0/4
			Low 1–10	0/1	0/10	0/6	0/1	
			Middle 10–100	1/0	3/4			
			High 100–1000		1/0	1/0		
			Highest > 1000					
		PCL-Gl-glu	0–1	0/15	0/2			
			Low 1–10	1/43	0/4			
			Middle 10–100	2/4	0/1			
			High 100–1000	2/0	1/0			
			Highest > 1000					
100	TCPS	PLGA	0–1	0/1	0/32	0/13		
			Low 1–10	0/2	0/15	0/2		
			Middle 10–100	3/3	2/1			
			High 100–1000		1/0			
			Highest > 1000					
		PCL	0–1	0/6	0/25	0/9	0/2	
			Low 1–10	2/22	0/15	0/2		
			Middle 10–100	3/7	3/2			
			High 100–1000					
			Highest > 1000	2/0				
		PCL-Gl	0–1	0/2	0/18	0/20	0/5	
			Low 1–10	2/20	0/13	0/2		
			Middle 10–100	3/8	3/1			
			High 100–1000	0/1				
			Highest > 1000	2/0				
		PCL-Gl-glu	0–1	0/26	0/1			
			Low 1–10	3/51				
			Middle 10–100	5/11	0/1			
			High 100–1000					
			Highest > 1000	2/0				
19	PCL-Gl-glu	PCL	0–1					
			Low 1–10	4/5				
			Middle 10–100	5/1				
			High 100–1000	3/1				

	Highest > 1000		
	0–1		
	Low 1–10	4/5	
PCL-Gl	Middle 10–100	4/1	1/0
	High 100–1000	3/1	
	Highest > 1000		
